# Dermatology

**Published:** 1906-03

**Authors:** W. Kenneth Wills


### DERMATOLOGY.

**Seborrhœa and Psoriasis.**—The connection between these disorders of the skin must appear evident to all dermatologists. Norman Walker[1] goes so far as to regard psoriasis as merely the extremely dry form of seborrhœa. It is not always evident as to what each writer may signify by his use of the term "seborrhœa"; but taking that it indicates what Sabouraud meant by it, viz. "flux of sebum," there must of necessity be a hyperactivity and hypertrophy of the sebaceous glands. In the condition known as seborrhœic dermatitis, however, the study of the histopathology leads Brimacombe to assert that there is a diminution of activity and sometimes an atrophy of the sebaceous glands, with an increase in size and function of the coil glands, the secretion being mainly sudoriparous.

This is compensatory for the loss of the oil glands, normal sweat containing oil. In psoriasis there is in addition to the disappearance of the sebaceous glands an atrophy of the coil glands, with a failure of the normal secretions of the skin affected. Brimacombe[2] has found a stage in seborrhœic dermatitis which histologically bears a close resemblance to incipient psoriasis, and suggests that the two affections bear the close connection the one with the other that Norman Walker holds in his view of the conditions.

**The treatment of lupus erythematosus,** or simply "erythematosus" which goes by the name of Holländer,[3] consisted in the administration of three grains of quinine daily, while tincture of iodine was painted on externally. This treatment has been somewhat modified by Moritz Oppenheim. After establishing the freedom from any idiosyncrasy he administers 0.50 gramme quinine morning and evening for three days, while the patches are cleansed with alcohol and ether and iodine painted on. Every third day of the administration 0.50 gramme is added to the dose, until eight half-grammes are being taken per diem. At this dose the patient remains until the foci of the disease are removed, and then the dose is decreased 0.50 gramme every

[1] Norman Walker, *Introduction to Dermatology*.
[2] *Lancet*, 1905, i. 419. [3] *Wien. Klin. Wchnschr.*, Jan. 19th, 1905.

third day. Only when tinnitus or deafness occurs is the treatment intermitted, and it is at once recommenced on the cessation of these symptoms. He claims that no untoward symptoms attend the use of these large doses of quinine; that in all cases tried he has had a successful result; that the treatment should be tried in all chronic cases; and that, inversely, the administration of iodides internally and quinine externally has no effect.

**The treatment of ringworm of the scalp by the X-rays** is now a recognised addition to the methods of attacking this common and intractable complaint. With the more accurate mensuration of the dosage permitted by Sabouraud's pastilles the danger of doing any harm is reduced to a minimum; and as far as has been recorded, no instance of a dermatitis sufficient to produce a cicatricial alopecia has resulted. The method aims at causing the hair in the affected areas to fall out after one application of the rays, and if this is successful, the use of some parasiticide to destroy the fungus which is not harmed by the rays in any way. In about three weeks the hair falls after a sufficient dose without more than a superficial erythema of a transitory nature. The hair grows again slowly, giving ample time for the effective destruction of the fungus, and in about four to six months the hair is restored completely. This method certainly has several advantages, not the least of which are the painlessness and the rapidity. Great care has to be exerted to ascertain that the treatment has been complete and that no stumps have been overlooked; individual stumps may be treated separately by the croton-oil needling method, but if the procedure recommended by Macleod[1] is adopted, the likelihood of many being left is reduced to a minimum.

As a means of preventing the lamentable interference with the education of children affected by tinea tonsurans, David Walsh[2] suggests the following treatment.

The scalp is shaved, rubbed over with turpentine, washed with soap and water, and then painted with a germicide, such as weak formalin. A ten per cent. solution of ac. salicyl. in collodion is then applied and repeated every second or third day, or sooner if there is any tendency to crack. After a week or ten days the collodion film is forced up by the growth of the hair. The collodion is then stripped off and the hairs come with it, diseased and healthy alike. In this way spread of the disease is prevented, the treatment is shortened, education is not interfered with, a cure can be looked for by systematic and recurrent removal of the hair: the treatment is practically limited to shaving and refilming the scalp every week or ten days, and in addition, if required, the X-rays can cautiously be used through the film.

**Syphilis.**—In May, 1905, Schaudinn and Hoffmann, of Berlin, announced their discovery **in syphilitic patients of a spirillum.**

[1] *Brit. M. J.*, 1905, ii. 13. [2] *Med. Brief*, Oct., 1905.

This they found not only in surface papules and chancres, but also in deeper tissues and in lymphatic glands. In twenty-eight cases examined the organism was found, and in eight of these it was seen in the glands. Schaudinn terms the organism he describes the "spirochæta pallida," to distinguish it from other similar organisms such as the "spirochæta refringens," which is a saprophyte constantly to be found in the stagnating secretions of lesions other than syphilis, and particularly about the genitals. It is stated that whereas the spirochæta refringens is alone found in lesions from non-specific sources, the spirochæta pallida is only to be found in the fresh juices of specific lesions, such as may be obtained from punctured glands or lesions of the skin. Another organism bearing resemblance to the spirochæta pallida is the trypanosoma equiperdum, which produces a disease in horses not altogether unlike syphilis in human beings.

The spirochæta pallida is described as follows:—

"Long, thin filamentous spiral or corkscrew-shaped organisms, with pointed ends. Length 4.10 mm., breadth at most 0.25 mm. The turns of the spiral number six to fourteen, averaging eight to ten: are narrow, regular and deep.

"Living, it is extremely delicate, weakly refractile and vigorously motile, progresses by rotating on its long axis, and when at rest shows undulatory movements in its whole length.

"In smears it is difficult to stain, and is seen with difficulty, very high powers being necessary for its detection." (Heidingsfeld and Markley.)[1]

So delicate an organism cannot be grown on artificial media, and as it cannot be tested according to Koch's postulates of specificity, no definite statement can be made as to the specific nature of this organism; but it is held that (1) it may be found constantly in untreated primary and secondary lesions, in the glands, and for short periods in the blood.

(2) It is not found in lesions of like character due to causes other than syphilis.

(3) It is found in the experimental lesions produced in apes by inoculation.

(4) It is not found in tertiary lesions, which are not infective.

Metchnikoff and Roux[2] have found the spirilla in twenty-three out of thirty-one monkeys inoculated with syphilis. They have also found them in secondary syphilitic papules in man (especially the younger papules), positions where the possibility of external contamination may be excluded. They were unable to find them in the non-specific lesions of acne, psoriasis, and scabies, and they conclude that they have sufficient grounds for believing that syphilis is a chronic spirillosis due to the spirochætæ described by Schaudinn. These observers never found the S. refringens in the monkeys, and hold that the fact that the S. pallida was not found in all their cases does not

[1] *Lancet-Clinic*, Nov. 25th, 1905. [2] *Ann. de l'Inst. Pasteur*, 1905, xix. 673.

disprove the specificity, as the failure to find the B. tuberculosis does not discount the part played in lupus by that organism.

Numerous observers have since been adding their testimony to the presence of a spirochæta answering to the above description in different stages of the disease.

It was found in the unbroken "pemphigus" bullæ on the palms and soles of a newly-born syphilitic infant (Levaditi). In this case none were found in the blood, which is but the vehicle of its transference; but Buschke and Fischer discovered it in the blood, during life, of a congenitally syphilitic infant.

In the primary indurated sores they have been found in not a few instances (McWeeney, Herxheimer and Hübner, Heidingsfeld and Markley, and others). McWeeney found them in secondary lesions, but could not find them in tertiaries. Jacquet and Sevier failed also to establish their presence in tertiary lesions. Leonard Dudgeon has found them in the fluid expressed from mucous patches; and Stachlein and Noegerath by diluting and centrifugalising blood from the lobe of the ear have found them in three cases of secondary syphilis, but they were unable to find them in healthy, unsyphilitic cases.

Heidingsfeld and Markley have found them in two cases of ulcus molle and in condylomata, as well as in primary and secondary lesions; and Kiolomeneglon and Cube state that they have found them not only in syphilitics but in balanitis, gonorrhœa, and in the decomposition products of carcinoma.

As time goes on other non-specific cases may be found to harbour these spirilla, and in any case it would be inadvisable to make any definite statement yet as to the specificity of the spirochæta pallida. The investigation of the whole group of these organisms is so recent, that it will be well to collect much information before taking so definite a stand as Metchnikoff and Roux. It has to be proved first that it is not a coincident saprophyte symbiotic with the real cause of syphilis.

**Syphilis and Yaws.**—As a corollary of more than usual interest, considering the closeness of resemblance between the two diseases, and the arguments for and against the two being identical, comes the announcement that Castellani[1] has found spirochætæ in yaws. He was unable to find these organisms in two cases of dry lesions, but in two ulcerated cases he found spirochætæ together with myriads of bacteria. In one case, a Cingalese lad of ten years, presenting no signs of congenital or acquired syphilis, was in the secondary stage of yaws. He had desquamation of the hands and arms, but the lower legs and soles presented several fungoid, roundish, elevated, slightly secretive formations. The spirochætæ were found in these ulcerated lesions. Although he found several varieties of spirochætæ, one variety which Castellani[2] submitted to Schaudinn was recognised by that authority as very closely resembling his spirochæta pallida.

[1] *Brit. M. J.*, 1905, ii. 1280. [2] *Ibid.*

This adds but one more to the **similarities between yaws and syphilis,** which Jeanselme[1] tabulates as follows:—

1. Pains in the bones and joints, which become worse at night.
2. Tendency towards a circular form in the eruptions.
3. Preference for the neighbourhood of orifices of the body.
4. The curative power of potassium iodide and mercury.

As Jeanselme points out, Charlonis proved they are not the same disease by successfully inoculating a person with syphilis who was already suffering from yaws. Though in some respects they resemble each other very closely, yet the chief differences may be classified in the following way:—

| SYPHILIS. | YAWS. |
|---|---|
| Pandemic, acquired by heredity and contagion. | Tropical, acquired by contagion only. |
| Begins by primary pathognomonic lesion at point of inoculation. | Initial lesions not constant and not differing from later appearances. |
| Immunity conferred by syphilis almost certain. | Re-infection possible. |
| Auto-inoculation impossible. | Auto-inoculation possible for a long and unknown length of time. |
| A hard chancre and other syphilitic signs can be found on patient who has suffered from yaws. | A syphilitic patient can become affected with yaws. |
| A great variety of eruptions. | Only one eruption, the papillomata. |
| The eruption, especially in tertiary stage, damages the skin, and leaves indelible scars after cure. | Eruption leaves no scar unless unduly irritated. |
| Three definite stages: Primary, secondary, tertiary. | All manifestations identical whatever date. |
| The mucous membrane may be involved. | Mucous membranes escape. |
| Internal organs may be involved. | No affection of internal organs. |
| No itching. | Much itching. |
| Hair falls in secondary stage. | Hair never falls out. |

W. KENNETH WILLS.

